# Inflammation and dyslipidaemia in combined diabetes and tuberculosis; a cohort study

**DOI:** 10.1016/j.isci.2025.112760

**Published:** 2025-05-27

**Authors:** Julia Brake, Mandala Ajie, Nicholas A. Sumpter, Raspati C. Koesoemadinata, Nanny N.M. Soetedjo, Prayudi Santoso, Bachti Alisjahbana, Rovina Ruslami, Philip Hill, Reinout van Crevel

**Affiliations:** 1 Department of Internal Medicine, Radboud University Medical Center, Nijmegen, the Netherlands; 2 Department of Internal Medicine, Leiden University Medical Center, Leiden, the Netherlands; 3 Research Center for Care and Control of Infectious Disease, Universitas Padjadjaran, Bandung, Indonesia; 4 Division of Endocrinology, Metabolic Disorders and Diabetes, Department of Internal Medicine, Padjadjaran University, Bandung, West Java, Indonesia; 5 Division of Respirology and Critical Care, Department of Internal Medicine, Faculty of Medicine, Universitas Padjadjaran and Hasan Sadikin General Hospital, Bandung, Indonesia; 6 Division of Pharmacology and Therapy, Department of Biomedical Sciences, Faculty of Medicine, Universitas Padjadjaran, Bandung, Indonesia; 7 McGill International TB Centre, McGill University, Montreal, QC, Canada; 8 Centre for International Health, Otago University, Dunedin, New Zealand; 9 Centre for Tropical Medicine and Global Health, Nuffield Department of Medicine, University of Oxford, Oxford, UK

**Keywords:** Health sciences, Medicine, Medical specialty, Immunology, Endocrinology, Medical microbiology

## Abstract

Diabetes mellitus (DM) increases tuberculosis (TB) susceptibility and worsens outcomes. Since inflammation and lipid metabolism are implicated in both diseases, we examined if combined TB and DM (TB-DM) increases inflammation or dyslipidaemia. In plasma from individuals with DM (*n* = 96), TB (*n* = 93), and TB-DM (*n* = 91), we measured 92 inflammatory proteins and 250 primarily lipid-related metabolites, repeating measurements after two months of TB treatment. Inflammation was primarily driven by TB, but higher in TB-DM. In TB-DM, the proteins osteoprotegerin (OPG), signaling lymphocytic activation molecule (SLAMF1), adenosine deaminase (ADA), interleukin-10 receptor subunit beta (IL-10RB), and tumor necrosis factor receptor superfamily member 9 (TNFSR9) were differentially abundant, and IL-17A/C predicted treatment failure. Disease severity correlated with inflammation and dyslipidaemia. Inflammation decreased with TB treatment, both in TB and TB-DM. Dyslipidaemia was primarily driven by DM, but more pro-atherogenic in TB-DM, with elevated VLDL and apolipoprotein B (ApoB). Despite TB treatment, pro-atherogenicity persisted. Stronger inflammation and dyslipidaemia may account for worse disease outcomes in TB-DM and warrant further action to prevent cardiovascular events.

## Highlights

• Combined tuberculosis and diabetes (TB-DM) increases inflammation and pro-atherogenic lipids
• Tuberculosis treatment decreases inflammation, but pro-atherogenic lipid profiles persist
• Pro-atherogenic lipid profiles warrant further action to prevent cardiovascular events
• Disease severity is linked to inflammation and dyslipidaemia

## Graphical abstract

**Figure:**
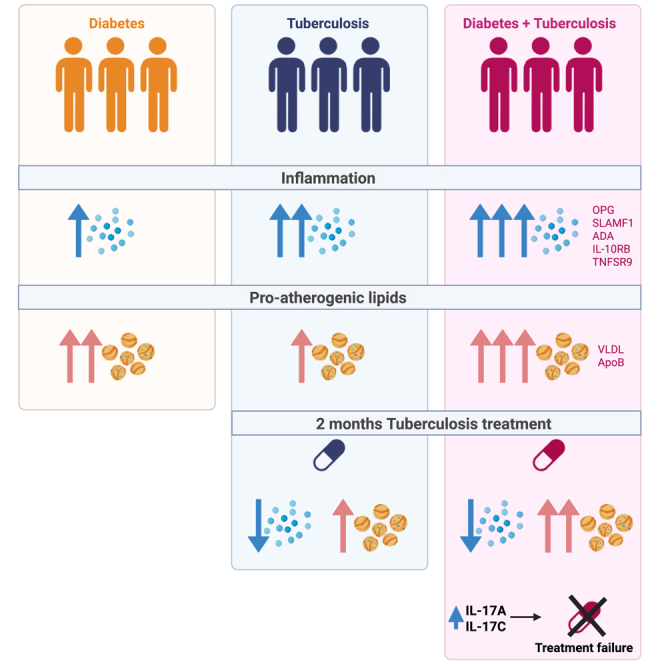

## Introduction

Combined diabetes mellitus (DM) and tuberculosis (TB) is associated with more severe disease and poor treatment outcomes.^1^ Type 2 DM triples the risk of developing active TB, and individuals with tuberculosis-diabetes comorbidity (TB-DM) exhibit a higher bacterial load,^2^ more pulmonary cavities,^3^ and severe dysglycemia.^4^^,^^5^ Moreover, those with TB-DM are twice as likely to die during TB treatment compared to individuals with TB but no DM.^4^ Our understanding of the biological mechanisms underlying increased morbidity and mortality in TB-DM is incomplete. This knowledge gap is increasingly relevant as the number of individuals with combined TB-DM increases in light of the continuing rise of diabetes in TB-endemic countries.^6^

Inflammation and lipid metabolism are known to play a role in both diseases. Both TB and DM are inflammatory conditions.^7^^,^^8^ In TB, pro-inflammatory cytokines, chemokines, and growth factors are essential components in the host response against *Mycobacterium tuberculosis* (*Mtb*),^8^^,^^9^ but excessive inflammation leads to more severe TB disease.^10^ In the context of DM, chronic low-grade inflammation is a hallmark of the disease and is linked to dysregulation of immune cells, including heightened activation of macrophages and neutrophils,^11^^,^^12^^,^^13^ which may also alter the course of TB infection.^14^ Lipids are not only known to be involved in the development of atherosclerosis but also play an important role in the outcome of infections.^15^ Dyslipidaemia is a characteristic of type 2 DM as well as TB.^16^ In DM, dyslipidaemia mainly results from increased VLDL-production in the liver as a consequence of increased hepatic fatty acid supply. This leads to elevated levels of triglycerides (TGs), low-density lipoprotein (LDL) particles, and decreased high-density lipoprotein (HDL) cholesterol.^17^ Conversely, people with TB show signs of wasting, characterized by reduced total lipid concentrations due to the high metabolic demands of chronic infection.^18^^,^^19^

Inflammation and dyslipidaemia may play a role in disease manifestation and outcome in TB-DM. Additionally, inflammatory proteins and lipids might predict disease outcomes and direct drug therapy. However, only a few studies have examined inflammation and lipids before and during TB treatment in TB-DM, and have shown some association between dyslipidaemia^16^^,^^20^ and altered inflammation^21^^,^^22^^,^^23^ with TB-DM. More comprehensive studies are needed, especially exploring changes over TB treatment. We hypothesized that in TB-DM, inflammation would be accelerated and lipid levels altered, even during TB treatment, which may relate to more severe disease manifestations and worse treatment outcomes.

## Results

### Clinical characteristics of study participants

This study included a total of 280 individuals, divided into those with DM (*n* = 96), TB (*n* = 93), and TB-DM (*n* = 91), with similar sex distribution between groups (Table 1). Individuals with type 2 DM alone had a median BMI of 25.2 kg/m^2^ and HbA1c of 9.3%. Most had been diagnosed for over six years prior to enrollment. One-fourth (27.1%) were prescribed insulin, 52.1% metformin, and 13.5% statins. Complications, such as ocular issues, were common (Table 1). Compared to those with TB, the DM-only group was significantly older and had a higher BMI, with 53.1% classified as obese (Table S2). All had been screened negative for TB using symptom screen, sputum culture, and chest X-ray. Half of the individuals with DM had a positive interferon gamma release assay (IGRA) test result, which did not affect the outcomes of this study. For individuals with TB but without DM, the median BMI was 18.8 kg/m^2^, HbA1c was 5.6%, and hemoglobin was 12.6 g/dL (Table 1). The group had an elevated median Timika chest X-ray severity score of 48.3, with 22 individuals (23.7%) scoring above 71, compared to 13 individuals (14.3%) in the TB-DM group. Resistance to isoniazid or the combination with rifampicin was found in four people in those with TB alone and in two people in those with TB-DM (deposited data). Participants with TB-DM had the highest HbA1c levels among all groups, with 56 individuals (61.5%) showing a HbA1c above 10%. The median BMI in this group was 21.3 kg/m^2^, and 17.6% had been diagnosed with DM more than six years previously. In terms of treatment, 34.1% were prescribed insulin, 82.4% metformin, 23.1% sulfonylureas, and 2.2% statins. Compared to those with TB alone, slightly more individuals with TB-DM remained sputum culture positive after 6 and 12 months of TB treatment (Table 1). Among those with TB-DM, none were HIV infected, and three individuals showed resistance to isoniazid (deposited data).

**Table 1:** Patient characteristics and treatment outcome

	DM (*n* = 96)^a^	TB (*n* = 93)^a^	TB-DM (*n* = 91)^a^
Age, years	54.0 [25.0, 77.0]	41.7 [18.9, 84.4]	50.3 [23.6, 75.1]
Female sex	39 (40.6%)	44 (47.3%)	41 (45.1%)
BMI, kg/m^2^	25.2 [16.6, 35.6]	18.8 [13.9, 33.3]	21.3 [14.6, 32.8]
Waist-to-hip ratio	0.92 [0.77, 1.06]	0.80 [0.68, 0.98]	0.87 [0.691, 1.05]
Females	0.88 [0.77, 0.98]	0.79 [0.68, 0.92]	0.85 [0.69, 0.97]
Males	0.93 [0.82, 1.06]	0.81 [0.70, 0.98]	0.87 [0.75, 1.05]
Smoking	23 (24.0%)	14 (15.1%)	12 (13.2%)

**Diabetes parameters**			

HbA1c, %	9.30 [6.60, 16.7]	5.60 [4.70, 5.90]	11.1 [6.60, 17.4]
DM duration			
<1 year	17 (17.7%)	NA	20 (22.0%)
1–5 years	38 (39.6%)	NA	29 (31.9%)
>6 years	41 (42,7%)	NA	16 (17,6%)
Medication^b^			
Insulin	26 (27.1%)	NA	31 (34.1%)
Metformin	50 (52.1%)	NA	75 (82.4%)
Sulfonylurea	0 (0%)	NA	21 (23.1%)
Statins	13 (13.5%)	NA	2 (2.2%)
Complications (history)			
Myocardial infarction	11 (11.5%)	NA	0 (0%)
Stroke	9 (9.4%)	NA	0 (0%)
Ocular problems or visual loss	48 (50%)	NA	4 (4.4%)
Kidney failure	2 (2.1%)	NA	0 (0%)

**Tuberculosis parameters**			

Anemia, g/dL^c^ (<11)	5 (5.2%)	17 (18.3%)	11 (12.1%)
Hemoglobin, g/dL	14.1 [7.90, 18.2]	12.6 [6.80, 15.8]	13.2 [7.30, 17.2]
Females	13.2 [7.90, 16.2]	11.9 [6.80, 14.3]	12.1 [8.80, 15.0]
Males	14.5 [9.50, 18.2]	12.9 [7.70, 15.8]	13.7 [7.30, 17.2]
Timika score	NA	48.3 [0, 123]	30.0 [0, 128]
Positive sputum culture^d^			
Month 2	NA	78 (83.9%)	69 (75.8%)
Month 6	NA	11 (11.8%)	16 (17.6%)
Month 12	NA	3 (3.2%)	5 (5.5%)
Month 18	NA	1 (1.1%)	1 (1.1%)
Death^e^	0 (0%)	4 (4.3%)	5 (5.5%)

a Summarized cohort characteristics based on patients, who are included in the inflammatory protein analysis. For lipid and metabolic intermediate analysis, less patients were included (DM = 93, TB = 91, TB-DM = 83). Data was incomplete for “Positive sputum culture” at month 2 (TB = 4, TB-DM = 2), at month 6 (TB = 2, TB-DM = 2), at month 12 (TB = 16, TB-DM = 5), at month 18 (TB = 26, TB-DM = 12), and for “Timika score” (TB = 6, TB-DM = 2). Data are presented as number or median [min, max]. TB, tuberculosis; DM, type 2 diabetes mellitus; TB-DM, tuberculosis-diabetes co-morbidity; BMI, Body mass index; HbA1c, glycated hemoglobin; NA, not applicable.

b Patients may be in more than one category.

c Moderate or severe anemia based on hemoglobin concentration.

d At least one positive sputum culture of *Mycobacterium tuberculosis* at indicated months after start of TB-treatment.

e Includes patients with TB, who died within 18 months after inclusion.

### TB-DM displays an accelerated inflammatory profile mainly driven by TB

**Figure 1:**
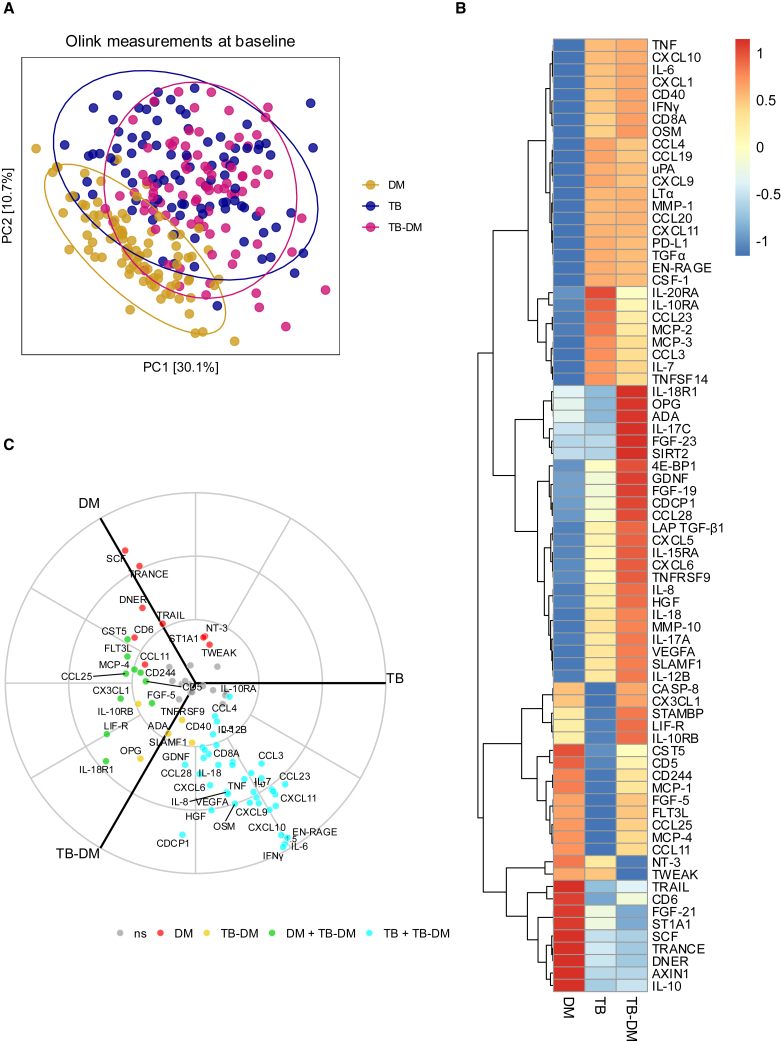
Inflammation at baseline Comparison of circulating inflammatory markers of individuals with DM (*n* = 96), TB (*n* = 93), and TB-DM (*n* = 91) at baseline. (A) Principal-component analysis (PCA) plot shows the first two principal components derived from individuals with DM (yellow), TB (blue), and TB-DM (pink). (B) Heatmap representing the z-scores of the median of the three groups for each measured circulating inflammatory marker. Higher and lower *Z* scores are depicted in red and blue, respectively. (C) Radial Volcano plot showing a cross-section of a three-dimensional Volcano plot comparing circulating inflammatory markers between the three groups. Circulating inflammatory markers, which are nonsignificant (gray), only significantly higher in DM (red), TB (not applicable), and TB-DM (yellow) and overlapping significance between DM and TB-DM (green) as well as TB and TB-DM (turquoise) are shown. The *z* axis is plotted as -log_10_(adjusted *p* value) and the polar coordinates display the relative mean level of the measured marker. Statistical testing was performed by Wilcoxon rank-sum test (two-sided) between two of the three groups and Kruskal-Wallis test to compare all three groups.

Proteomics analysis was used to measure circulating inflammatory markers in individuals with DM, TB, and TB-DM. Inflammatory protein profiles were more similar between people with TB and TB-DM in comparison to people with DM, suggesting that inflammation is primarily driven by TB (Figure 1A). Plasma from individuals with TB-DM and TB displayed multiple comparable protein clusters that were not seen in those with DM alone, for example the first 20 proteins listed from top to bottom in Figure 1B (TNF to CSF-1) showed quite similar abundance. However, a few proteins appeared to be differentially abundant between TB and TB-DM, such as a cluster of six proteins (interleukin-18 receptor 1 [IL-18R1], osteoprotegerin [OPG], adenosine deaminase [ADA], IL-17C, fibroblast growth factor 23 [FGF-23], and sirtuin-2 [SIRT2]), which was especially elevated in TB-DM (Figure 1B). At an individual protein level, a higher abundance in inflammatory protein levels was seen in TB and TB-DM compared to DM, such as for interferon gamma (IFNγ), interleukin-6 (IL-6), extracellular newly identified receptor for advanced glycation end-products binding protein (EN-RAGE), and C-X-C motif chemokine 10 (CXCL10), while some other proteins (e.g., SCF, TNF-related activation-induced cytokine (TRANCE), delta and notch-like epidermal growth factor-related receptor [DNER], and TNF-related apoptosis-inducing ligand (TRAIL) showed the highest abundance in DM. No proteins were found to be only specifically elevated in individuals with TB alone, while five proteins (OPG, ADA, SLAMF1, IL-10RB, and TNFRSF9) were significantly higher in TB-DM compared to DM and TB alone (Figure 1C). Additionally, when comparing only TB and TB-DM, IL-18R1, LIF-R, CDCP1, OPG, CX3CL1, and IL-10RB among others emerged as being significantly higher in TB-DM, suggesting accelerated inflammation in TB-DM compared to TB (Figure S1C). Results were barely affected by correction for age, sex, or BMI (Figures S2 and S3).

### TB treatment reduces inflammation in both TB and TB-DM

**Figure 2:**
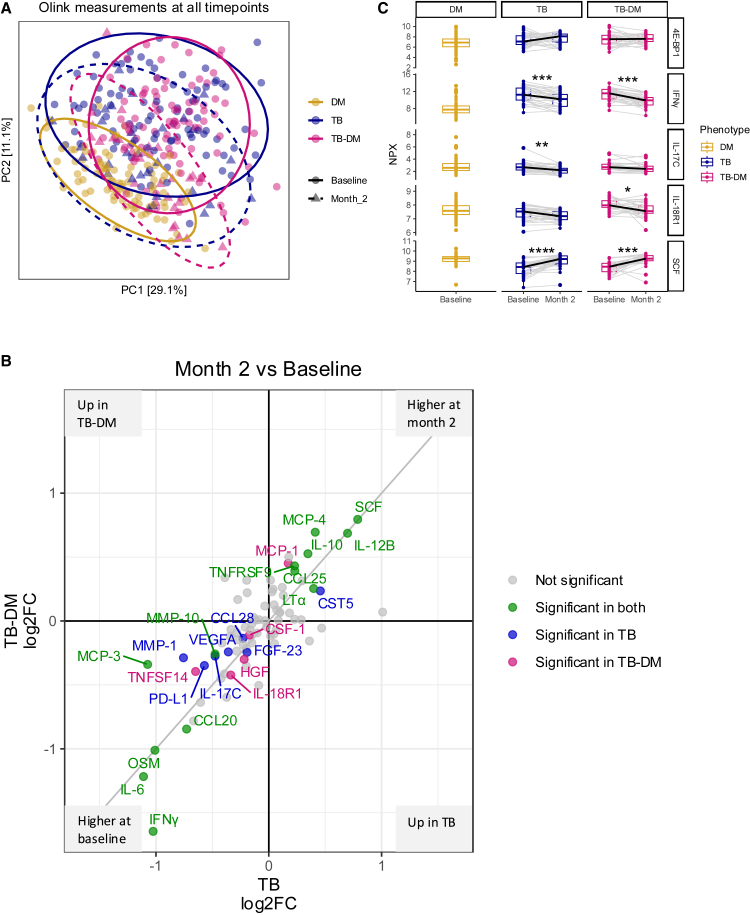
Inflammation after two months of TB treatment Paired changes of circulating inflammatory markers after two months of TB treatment in individuals with TB (*n* = 31) and TB-DM (*n* = 33) are displayed. (A) Principal-component analysis plot showing principal component one and two for individuals with DM (yellow), TB (blue), and TB-DM (pink) at baseline and for TB (blue, triangles) and TB-DM (pink, triangles) after two months of TB treatment. (B) Combined Volcano plot displaying the Log_2_ fold change between baseline and after two months of TB treatment for TB and TB-DM. Significant changes between two months TB treatment and baseline in both groups (green), in TB alone (blue), in TB-DM alone (pink), and nonsignificant changes (gray) are displayed. Statistical testing was done by paired Wilcoxon ranked-sum test (adjusted *p* value <0.05). (C) Boxplots showing the median plus interquartile ranges of normalized protein expression (NPX) for 4E-BP1, IFNy, IL-17C, IL-18R1, and SCF as examples for changing marker levels after two months of TB treatment. Statistical testing was done by paired Wilcoxon ranked-sum test (adjusted *p* value <0.05). ∗ adj. *p* < 0.05, ∗∗ adj. *p* < 0.01, ∗∗∗ adj. *p* < 0.001.

To examine if resolution of inflammation following TB treatment is impaired in TB-DM, we next compared inflammatory protein levels before and after two months of TB treatment. After two months of therapy, protein profiles of participants with TB and TB-DM appeared to shift closer to those of individuals with DM, reflecting an overall decrease in inflammation (Figure 2A). For example, IFNγ, IL-6, and oncostatin-M (OSM) were clearly elevated before start of treatment and decreased by more than 2-fold after two months of TB treatment (Figure 2B). SCF was significantly heightened after two months of TB treatment compared to baseline. Some proteins showed a phenotype-specific response to TB treatment. In particular, plasma IFNγ showed a stronger reduction in people with TB-DM than in those with TB only. Also, 4E-BP1 increased by 2-fold after two months of TB treatment among people with TB but barely changed in those with TB-DM (Figures 2B-2C). Some other inflammatory proteins showed phenotype-specific changes upon TB treatment. For example, IL-18R1 decreased in TB and TB-DM but more strongly in TB-DM. IL-18R1 was shown to be the only significantly elevated protein in TB-DM, when comparing only TB-DM and TB after two months of TB treatment (Figure S1D), which was non-significant after correcting for age, sex, or BMI (Figures S2D and S3D). A change towards the protein levels in people with DM could be observed for multiple displayed proteins (e.g., IFNγ and SCF), indicating once more a decrease of inflammation (Figure 2C). Overall, pro-inflammatory markers decreased in a similar way in TB and TB-DM upon TB treatment, while some proteins showed phenotype-specific alterations.

### Inflammation is associated with TB-severity and treatment failure

**Figure 3:**
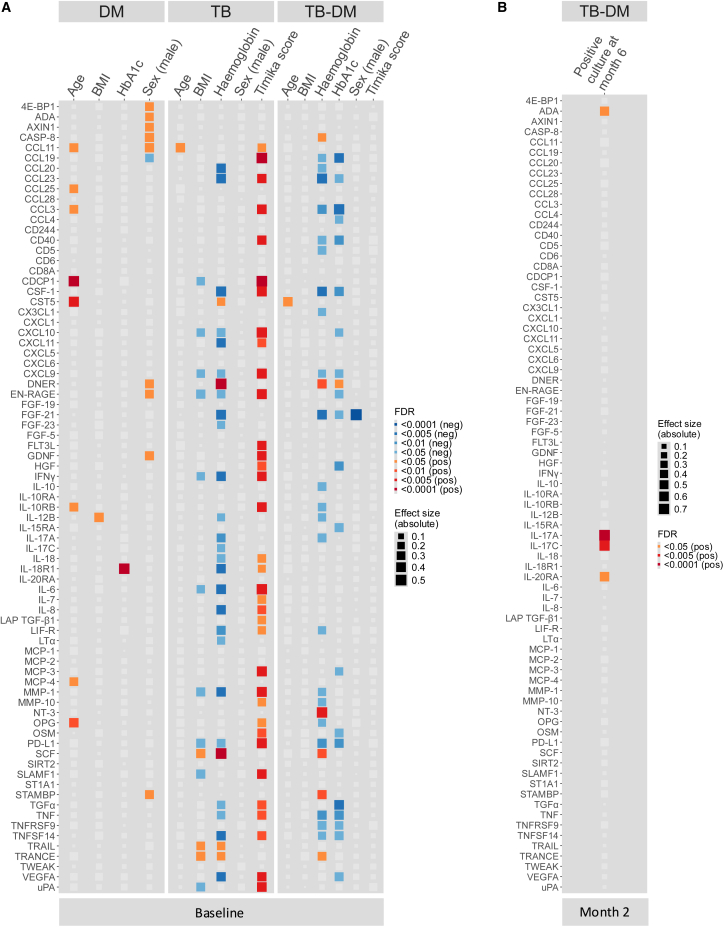
Associations of inflammatory proteins with clinical characteristics and treatment outcome (A) Linear regressions and absolute effect sizes representing the association between baseline circulating inflammatory markers and age, sex, BMI, HbA1c, hemoglobin, and Timika score for individuals with DM (*n* = 96), TB (*n* = 93), and TB-DM (*n* = 91). (B) Linear regressions and absolute effect sizes representing the association between circulating inflammatory markers after two months of TB treatment and a positive sputum culture result after six months of TB treatment representing treatment failure in individuals with TB-DM (negative sputum culture at month two [*n* = 28], positive sputum culture at month two [*n* = 3]). (A and B) Negative and positive associations are depicted in blue and red, respectively (FDR <0.05). The absolute effect size is depicted by the size of the square. All variables were tested independently by linear regression.

We next correlated inflammatory protein levels to clinical characteristics. In people with DM, some baseline inflammatory protein levels correlated positively with age and male sex (Figure 3A). DM duration was not associated with inflammatory markers (data not shown). IL-18R1 strongly correlated with HbA1c and IL-12B with BMI. In people with TB, we found a negative association for 10 proteins with BMI and a positive correlation of 35 plasma proteins with Timika score as a marker of chest X-ray severity (with low BMI and more severe X-rays reflecting advanced disease). Noteworthy, proteins that inversely correlated with BMI were similar to the proteins, which showed a positive correlation with the Timika score. In TB-DM, higher levels of inflammatory proteins (20 proteins) correlated with lower HbA1c levels, which are linked to hemoglobin levels. Somewhat surprisingly, no association was found with the Timika score. Both among people with TB and TB-DM, higher levels of many inflammatory proteins correlated with low hemoglobin levels, with much overlap between groups, while some proteins (e.g., DNER, SCF, and TRANCE) showed a positive correlation with hemoglobin concentration (Figure 3A). A subset of individuals with TB or TB-DM was followed for 18 months after start of TB treatment, and sputum was collected for *Mtb* cultures. In individuals with combined TB-DM, from whom none harbored a drug-resistant *Mtb* strain, higher levels of four inflammatory proteins (ADA, IL-17A, IL-17C, and IL-20RA) after two months of TB treatment significantly predicted positive *Mtb* sputum culture results after six months of TB treatment, with IL-17A and IL-17C most strongly associated. This was not found for individuals with TB alone (Figure 3B).

### Dyslipidaemia in TB-DM is pro-atherogenic and mainly driven by DM

**Figure 4:**
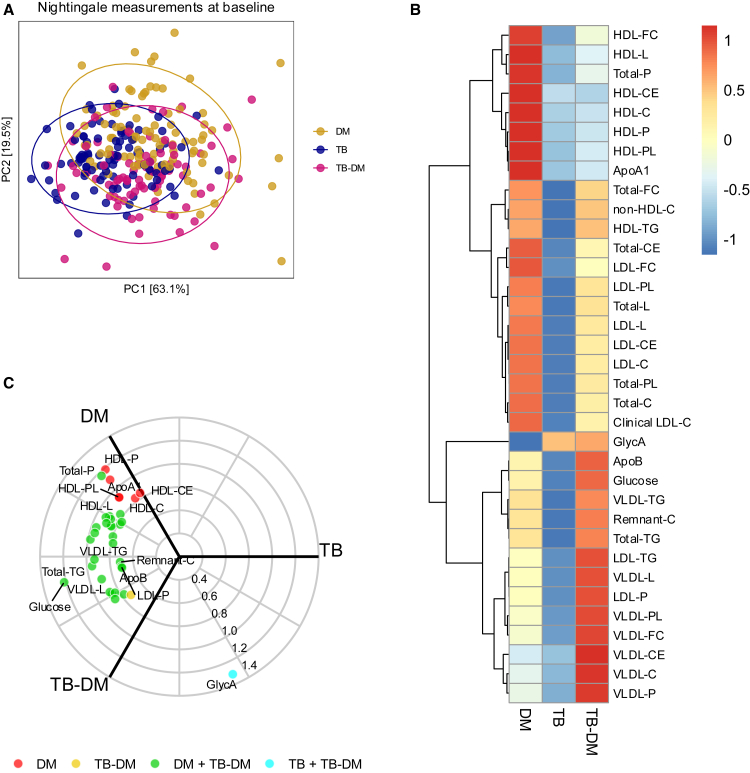
Lipids at baseline Comparison of lipid intermediates and metabolic markers of individuals with DM (*n* = 93), TB (*n* = 91), and TB-DM (*n* = 83) at baseline. (A) Principal Component Analysis (PCA) plot shows the first two principal components derived from individuals with DM (yellow), TB (blue), and TB-DM (pink). (B) Heatmap representing the *Z* scores of the median of the three groups for each measured marker. Higher and lower *Z* scores are depicted in red and blue, respectively. (C) Radial Volcano plot showing a cross-section of a three-dimensional Volcano plot comparing Lipid intermediates and metabolic markers between the three groups. Markers, which are nonsignificant (gray), only significantly higher in DM (red) or TB-DM (yellow) and overlapping significance between DM and TB-DM (green) as well as TB and TB-DM (turquoise) are shown. The *z* axis is plotted as -log_10_(adjusted *p* value) and the polar coordinates display the relative mean level of the measured marker. Statistical testing was performed by Wilcoxon rank-sum test (two-sided) between two of the three groups and Kruskal-Wallis test to compare all three groups.

Measurements of lipid and other metabolic intermediates at baseline revealed a partial overlap between the three groups, with slightly more similarity between TB-DM and DM (Figure 4A). At an individual level, most lipids showed more similar plasma levels between TB-DM and DM (Figure 4B). However, HDL, an anti-atherogenic lipoprotein, showed more similarity between TB-DM and TB and was higher in people with DM, while VLDL and apolipoprotein B (ApoB), pro-atherogenic proteins, were most elevated in TB-DM. GlycA, a marker for systemic inflammation, was higher in TB and TB-DM compared to DM, and, as expected, glucose was lowest in individuals with TB but without DM (Figure 4B). A significantly higher abundance of all lipid-related metabolites was seen in TB-DM and DM compared to TB, while some lipid intermediates (ApoA1, HDL-P, HDL-PL, HDL-C, and HDL-CE) were found to be only specifically elevated in DM (Figure 4C). VLDL-CE was specifically increased in people with TB-DM (Figure 4C), and when compared to DM alone, all VLDL’s were significantly increased in TB-DM, and ApoB, a predictor of atherosclerotic risk, was slightly elevated, while most HDL’s and LDL’s were significantly higher in DM (Figure S1F). In summary, TB-DM was associated with high lipid levels, especially of pro-atherogenic lipids VLDL and ApoB. Results were barely affected by correction for age, sex, or BMI (Figures S2 and S3). Additional metabolic measurements can be found in Figure S5.

### TB-DM is associated with pro-atherogenic lipid profiles during TB treatment

**Figure 5:**
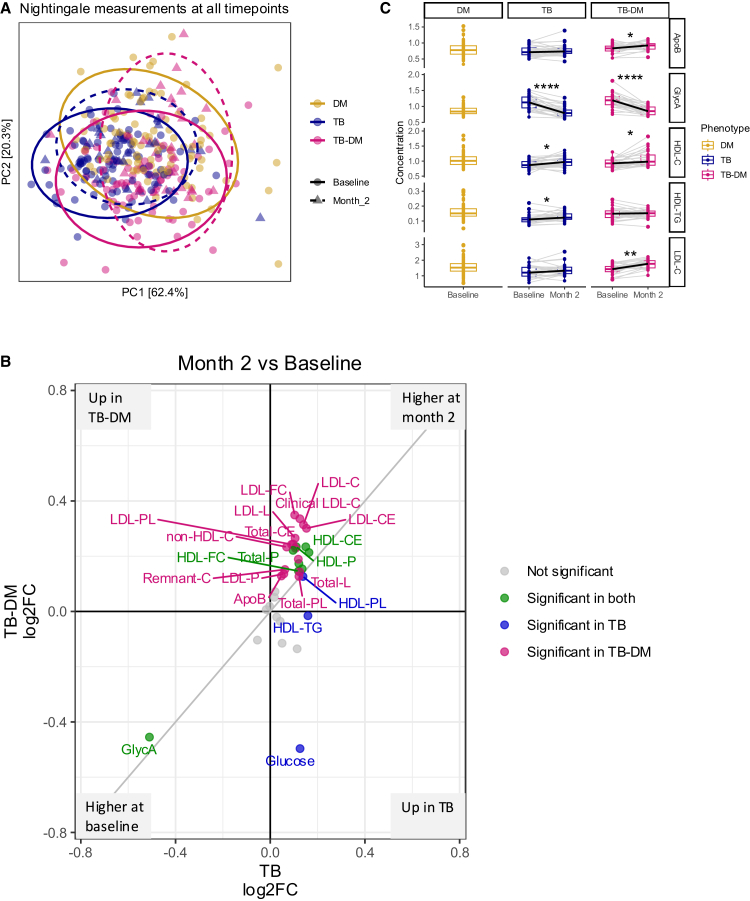
Lipids after two months of TB treatment Paired changes of lipid intermediates and metabolic markers after two months of TB treatment in individuals with TB (*n* = 32) and TB-DM (*n* = 31) are displayed. (A) Principal-component analysis plot showing principal component one and two for individuals with DM (yellow), TB (blue), and TB-DM (pink) at baseline and for TB (blue, triangles) and TB-DM (pink, triangles) after two months of TB treatment. (B) Combined Volcano plot displaying the Log_2_ fold change between baseline and after two months of TB treatment for TB and TB-DM. Significant changes between two months TB treatment and baseline in both groups (green), in TB alone (blue), in TB-DM alone (pink), and nonsignificant changes (gray) are displayed. Statistical testing was done by paired Wilcoxon ranked-sum test (adjusted *p* value <0.05). (C) Boxplots showing the median plus interquartile ranges of the plasma concentration for ApoB (g/L), GlycA (mmol/L), HDL-C (mmol/L), HDL-TG (mmol/L), and LDL-C (mmol/L) as examples for changing marker levels after two months of TB treatment. Statistical testing was done by paired Wilcoxon ranked-sum test (adjusted *p* value <0.05). ∗ adj. *p* < 0.05, ∗∗ adj. *p* < 0.01, ∗∗∗ adj. *p* < 0.001.

We then investigated whether dyslipidaemia resolves with TB treatment. Among individuals with TB and TB-DM, lipid profiles seemed to be modified after two months of TB treatment (Figure 5A). Most lipid-related metabolites had slightly increased in TB and TB-DM, with higher log2 fold changes in TB-DM (Figure 5B). Especially LDL’s and ApoB only showed a significant elevation in TB-DM. GlycA, a marker of systemic inflammation, showed a similar downward trend in TB and TB-DM (Figures 5B-5C). Glucose was higher in TB-DM at baseline but decreased upon TB treatment (Figure 5B). However, HDL-TG and HDL-PL changed only significantly in TB (Figures 5B-5C). On the whole, plasma lipid levels rose upon TB-treatment, while some lipids, such as LDL’s and the pro-atherogenic ApoB, showed a stronger increase in TB-DM compared to TB.

### Disease severity is associated with dyslipidaemia

**Figure 6:**
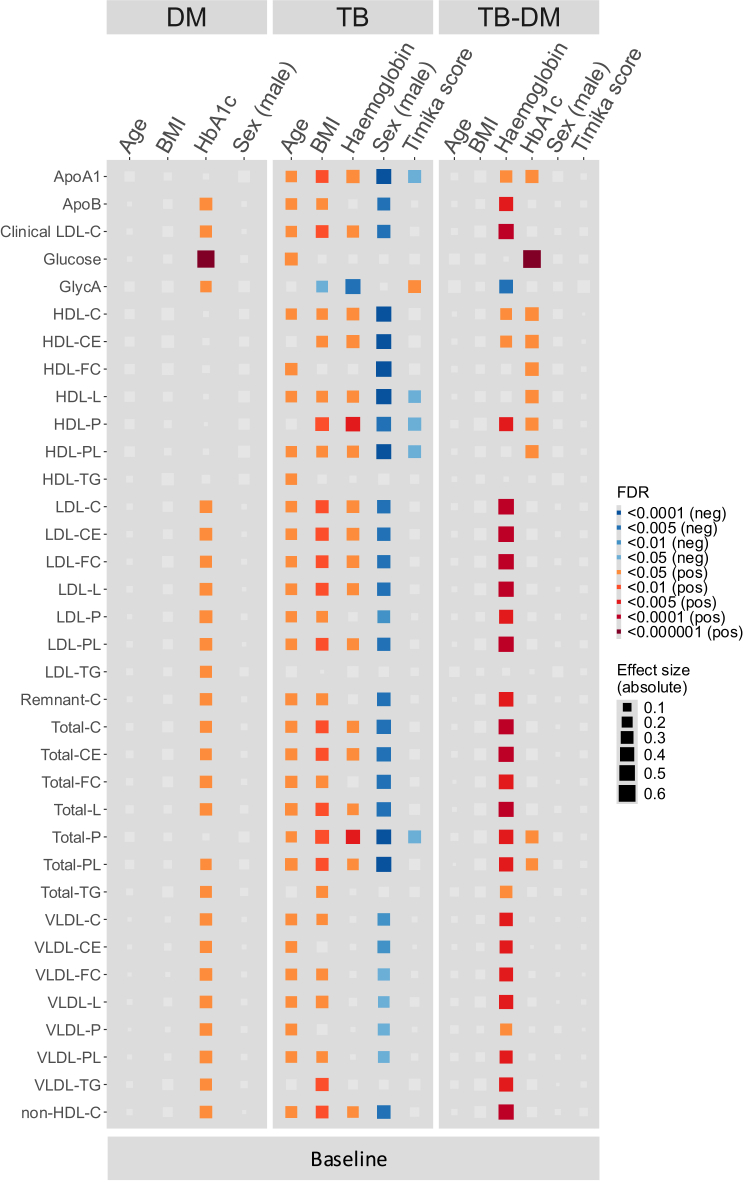
Associations of lipid markers with patient characteristics Linear regressions and absolute effect sizes representing the association between baseline circulating lipid intermediates and metabolic markers and age, sex, BMI, HbA1c, hemoglobin, and Timika score for individuals with DM (*n* = 93), TB (*n* = 91), and TB-DM (*n* = 83). Negative and positive associations are depicted in blue and red, respectively (FDR <0.05). The absolute effect size is depicted by the size of the square. All variables were tested independently by linear regression.

Next, we analyzed the associations of patient characteristics with lipid measurements. In people with DM, we found a positive association with HbA1c and 24 lipid intermediates, including LDL’s, VLDL’s, and total lipid abundances (Figure 6). Moreover, GlycA and glucose were positively associated with HbA1c. No associations were found with DM duration (data not shown). In people with TB, age, BMI, and hemoglobin were positively associated with 28, 28, and 18 lipid intermediates, respectively. Furthermore, we found a negative association of the Timika score with ApoA1, three HDL’s, and total phospholipids, but a positive association with GlycA. Participants with TB-DM showed a positive association with hemoglobin levels and 28 lipid intermediates. HbA1c was positively correlated with nine lipid intermediates and glucose. These lipid intermediates mainly included HDL’s. In people with TB and TB-DM, GlycA was negatively associated with hemoglobin (Figure 6). Overall, disease severity was associated with dyslipidaemia, which was reflected by HbA1c in DM and by hemoglobin levels in those with TB.

### Inflammatory markers and lipids are linked

**Figure 7:**
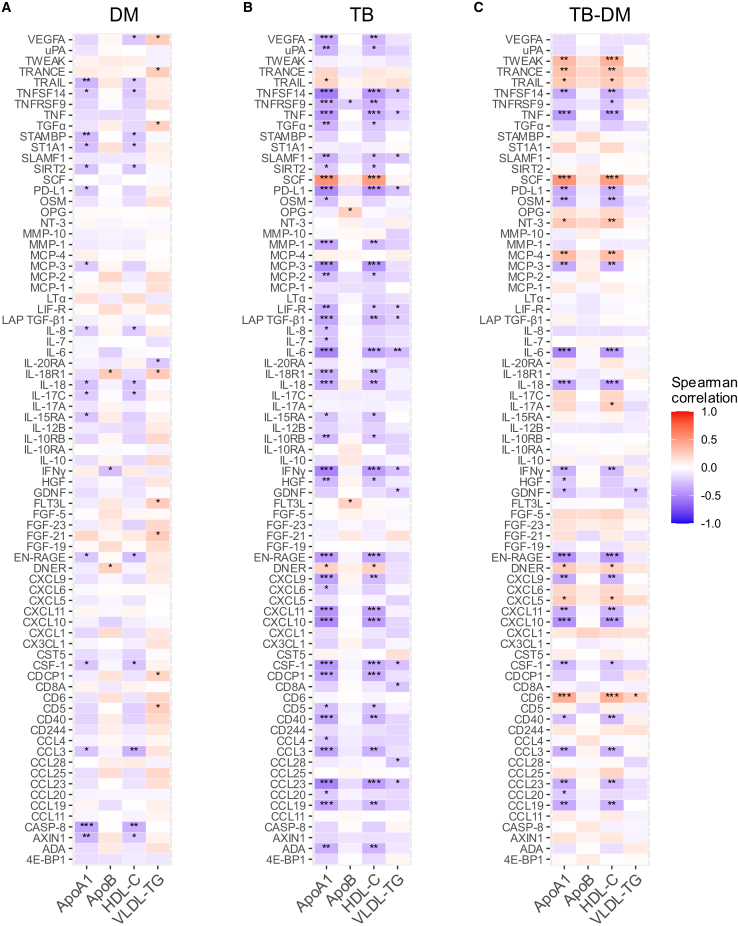
Correlation between inflammatory and lipid markers Heatmap displaying the spearman’s rank correlation coefficient between absolute lipid intermediate concentrations and normalized protein expression (NPX) values of circulating inflammatory markers for individuals with (A) DM (*n* = 92), (B) TB (*n* = 90), and (C) TB-DM (*n* = 82). Positive and negative correlation coefficient are depicted by red and blue, respectively (FDR <0.05).

Finally, we analyzed the relationship between inflammatory markers and a specific subset of lipid measurements for each phenotype. Overall, higher levels of inflammatory proteins correlated with lower levels of two anti-atherogenic lipids, ApoA1 and HDL cholesterol, with the exception of for example SCF and DNER (Figure 7). The inverse correlation between inflammatory proteins and anti-atherogenic lipids was most pronounced in TB only (Figure 7B). Individuals with combined TB and DM showed more positive correlations between anti-atherogenic lipids and inflammatory proteins, such as tumor necrosis factor (TNF)-family proteins (TWEAK and TRANCE) and the monocyte chemoattractant MCP-4 (Figure 7C). Inflammatory proteins mostly showed a positively correlation with the pro-atherogenic lipids ApoB and VLDL-TG in DM, but not in TB, and showed both positive and negative correlations in TB-DM (Figure 7).

## Discussion

We compared plasma inflammatory proteins and lipid profiles in people with TB, DM, and combined disease. Inflammation was primarily driven by TB, but higher in individuals with TB-DM compared to those who only had TB. Dyslipidaemia, on the other hand, was primarily driven by DM, but individuals with combined disease showed more pronounced pro-atherogenic lipid profiles. Finally, people with TB-DM showed a phenotype-specific correlation between plasma lipids and inflammatory markers and persistent pro-atherogenic lipid profiles during TB treatment.

Circulating inflammatory proteins were most abundant in TB-DM, more than in TB and especially more than in DM. Resolution of inflammation upon TB treatment followed a similar pattern in TB and TB-DM, with a few exceptions, such as 4E-BP1. Several other studies have found that inflammation is increased in TB and TB-DM^22^^,^^24^ and correlates with TB severity.^25^ Similar to our study, a Brazilian cohort showed increased baseline inflammation in TB-DM compared to TB only,^23^ which, differently from ours, persisted after two months of TB therapy (reanalyzed in Figure S4).

There are several factors that may account for higher baseline circulating inflammatory markers in TB patients with DM versus those without DM. Those with DM were older and had a higher BMI than individuals with TB. On the other hand, average TB-severity was lower in TB-DM, possibly because some individuals were actively diagnosed with early TB in DM-clinics. In regard to this, increased inflammation in TB-DM likely reflects chronic low-grade inflammation, which characterizes DM, combined with strong inflammation in the context of active TB. Hence, the combination of DM with TB accelerates inflammation. Most elevated inflammatory markers in TB-DM overlapped with TB and some with DM, showing that the inflammatory profile displays characteristics of both diseases. However, a few markers were specific for TB-DM, which might direct further study on the intersection of inflammatory responses in TB-DM. For instance, OPG is an independent biomarker for cardiovascular disease (CVD) and is elevated in people with DM and with cardiovascular and other complications,^26^ as well as worse glycemic control.^27^ This marker and others correlated with a more severe DM phenotype in individuals with TB-DM.

Inflammatory protein signatures as found in TB^28^ were quite similar in TB-DM, indicating that TB is the driving factor of inflammation in TB-DM. Additionally, while the duration of DM was shorter, inflammation was higher in individuals with combined TB-DM compared to those with DM alone. The most pronounced circulating inflammatory markers (IFNγ and IL-6) in TB-DM and TB are well-known mediators in TB. IFNγ activates macrophages and enhances the killing of *Mtb*.^29^ IL-6, produced by *Mtb*-infected macrophages, promotes differentiation of naive CD4^+^ T cells into Th17 cells while inhibiting transforming growth factor β (TGF-β)-induced Treg differentiation.^30^ Therefore, upregulation can disrupt immunological tolerance, and high levels of IL-6 are associated with TB progression.^31^ Although pro-inflammatory cytokines are meant to protect against *Mtb*, excessive inflammation in TB-DM might lead to immunopathology.

We found some specific markers that correlated with treatment failure. Elevated ADA, IL-17A, and -C at month two of TB therapy were associated with treatment failure in TB-DM. IL-17A is a pro-inflammatory cytokine secreted by γδ T cells and CD4^+^ Th17 cells. IL-17A initiates inflammation, particularly in the lung, and recruits and maintains the survival of macrophages^32^ and neutrophils,^33^ while contributing to mature granuloma formation in TB.^34^ However, prolonged IL-17 exposure of neutrophils has been associated with immunopathology,^35^ which is more common in TB-DM. Furthermore, an exaggerated IL-17A and Th17 cell response has been linked to increased neutrophil recruitment and elevated matrix metalloproteinase-1 expression in active TB, both of which play a role in TB pathogenesis, suggesting a more pronounced pathogenesis in combined TB-DM.^36^

With regard to plasma lipids, combined TB-DM was associated with pro-atherogenic lipid profiles, more similar to those of individuals with DM rather than TB alone. Particularly noteworthy was the observation that VLDL and ApoB levels, markers for increased atherosclerotic risk, were higher in TB-DM compared to DM or TB alone. Notably, we showed that TB treatment does not normalize pro-atherogenic lipid profiles in TB-DM, which suggests that individuals with combined TB-DM have increased risks of post-TB cardiovascular disease and mortality after successful completion of TB therapy.^37^

In individuals with type 2 DM or TB-DM, more TGs are stored in adipose tissue. In response to increased TG availability, the liver produces more VLDL, which contributes to heightened atherosclerotic risk.^38^^,^^39^^,^^40^ TGs, predominantly transported by VLDL, are exchanged with cholesterol esters from HDL via the cholesteryl ester transfer protein.^38^ Therefore, HDL-C levels are often reduced in DM, serving as an indicator of elevated VLDL levels. In our study, individuals with TB-DM exhibited lower HDL-C levels than people with DM alone, while they had the highest VLDL levels, indicating increased lipolysis and fatty acid flow to the liver.^38^ Both VLDL and LDL particles can penetrate the vessel wall and promote plaque formation, thereby increasing the risk of cardiovascular events.^38^ ApoB represents the total number of VLDL, IDL, and LDL particles, with each of these particles containing one structural ApoB protein. Therefore, ApoB serves as a robust predictor of cardiovascular risk.^41^ Individuals with TB-DM exhibited the highest levels of ApoB, suggesting that they have particularly pro-atherogenic lipid profiles and higher risks of cardiovascular events compared to those with DM alone.

Similar to our findings, a study from South Africa^16^ found that lipid levels were higher in individuals with TB-DM (*n* = 27) compared to TB alone (*n* = 50) and higher in individuals with DM (*n* = 50) compared to TB (reanalyzed in Figure S4). In addition to those data from South Africa, we observed a more pro-atherogenic lipid profile in individuals with combined TB-DM compared to individuals with DM alone, possibly due to the fact that combined TB-DM showed worse glycemic control compared to DM in our study, but not in the South African cohort.

Importantly, TB treatment did not normalize pro-atherogenic lipid profiles in TB-DM. Wasting and loss of appetite are well-known characteristics of TB^18^ and known to be associated with low LDL levels, as in other severe illnesses.^42^ Upon TB treatment, LDL levels increased, probably as a result of weight gain and resolving inflammation. Notably, pro-atherogenic ApoB, which was already higher in TB-DM at baseline compared to DM and TB only, showed a stronger increase upon treatment than in TB alone. This finding may account for the increased (early) mortality reported in TB-DM,^43^ especially among people who smoke.^44^ Our findings support the use of lipid-lowering drugs, such as statins, in TB-DM.^45^

We also examined correlations between lipid and inflammatory markers. In individuals with DM, inflammation was mostly associated with higher levels of the pro-atherogenic lipids ApoB and VLDL-TG, aligned with established links between chronic inflammation, dyslipidaemia and elevated CVD risk.^17^ Conversely, in TB patients, inflammation inversely correlated with pro-atherogenic lipids, probably because of TB-associated wasting.^16^^,^^18^ In individuals with combined TB and DM, the picture was mixed, reflecting pro-atherogenic lipids and elevated CVD risk driven by DM, and heightened inflammation associated with TB.

In conclusion, our study uncovers new associations between TB-DM, inflammation, and dyslipidaemia both before and after two months of TB treatment, using, to our knowledge, the largest TB-DM cohort studied in this context. Our findings regarding circulating inflammatory markers and lipids may help explain more severe disease manifestations of TB-DM, and underline recommendations to consider statins for TB-DM patients, especially those with prior cardiovascular disease.^45^

### Limitations of the study

This study has several limitations. Firstly, there was no cohort without DM or TB available for comparison. Although our primary focus was the combination of TB-DM (compared to TB and DM only), the lack of a healthy control group implicates that conclusions can only be drawn relative to the disease phenotypes studied. However, baseline differences between healthy individuals and those with DM, TB, or TB-DM can be inferred from previous studies,^16^^,^^46^ but direct comparisons might be compromised for instance due to varying study circumstances. Secondly, we did not adjust our findings for co-medication like metformin or statins, drugs which can affect inflammatory and lipid profiles. This subgroup analysis would lead to very small groups and statins are rarely used in this setting,^47^ and unfortunately, routine data on prescription and actual intake of these drugs were not complete. Also, adherence to medication was most likely not optimal regarding the observed high HbA1c and lipid levels in those who reportedly used glucose-lowering drugs and statins. Finally, the study was conducted in a single geographical setting. This limits generalizability and suggests that studies should be done in different populations. While studies about lipid profiles in an African cohort^16^ and inflammatory marker profiles in a Dutch cohort^46^ with healthy participants exist and our results have been partly validated in a South African^16^ and Brazilian^23^ cohort, future research should explore how these findings generalize to patient populations with greater ethnic diversity.

## Resource availability

### Lead contact

Further information and requests for resources should be directed to and will be fulfilled by the lead contact, Julia Brake (Julia.Brake@radboudumc.nl).

### Materials availability

This study did not generate new unique reagents.

### Data and code availability

• Original code and additional analyses have been deposited at GitHub as https://htmlpreview.github.io/?https://github.com/juliabrake/TANDEM/main/Analysis_Julia.html and are publicly available as of the date of publication.
• Olink and Nightingale data have been deposited at GitHub as https://github.com/juliabrake/TANDEM (TANDEM_Olink_data.xlsx and TANDEM_Nightingale_data.xlsx) and are publicly available as of the date of publication.
• Any additional data reported in this paper will be shared by the lead contact upon request.

## Acknowledgments

This study was supported by the ECFP7 (European Union’s Seventh Framework Programme) -funded consortium TANDEM (Tuberculosis and Diabetes Mellitus; grant number 305279) and by the EU-funded consortium PROTID (Prevention of Tuberculosis in Diabetes; grant number RIA2018CO-2514). The funder had no role in the study design, data collection and analysis, decision to publish, or preparation of the manuscript.

We thank the members of the TANDEM consortium and all study participants. We would like to thank Liesbeth van Emst for performing the Olink measurements in-house and Rinke Stienstra, Frank Vrieling, and Niels Riksen for discussing the data. The graphical abstract was created in BioRender. Brake, J. (2025) https://Biorender.com/u9dnqdb.

## Author contributions

Conceptualization and design of the TANDEM study, R.v.C., P.H., B.A, and R.R.; participant inclusion and sample collection, R.C. K., N.N.M.S., and P.S.; experimental design and sample selection, M.A. and J.B.; data analysis, J.B. and N.A.S.; data interpretation, J.B.; writing original draft, J.B.; review and editing, R.v.C.; reading and approval of final version, all authors. All authors had full access to the data and were responsible for deciding to submit it for publication.

## Declaration of interests

The authors declare no competing interests.

## STAR★Methods

### Key resources table

**Table:** 

REAGENT or RESOURCE	SOURCE	IDENTIFIER
**Biological samples**		

Human plasma samples	TANDEM cohort	https://doi.org/10.1016/S2213-8587(14)70011-7

**Critical commercial assays**		

Olink Target 96 Inflammation panel	Olink	https://olink.com/products/olink-target-96
Nightingale biomarker platform	Nightingale Health	https://research.nightingalehealth.com/

**Deposited data**		

Code for data analysis including quality controls and additional analyses	This paper; GitHub	https://htmlpreview.github.io/; https://github.com/juliabrake/TANDEM/main/Analysis_Julia.html
Olink target Inflammation panel data	This paper; GitHub	https://github.com/juliabrake/TANDEM/blob/main/TANDEM_Olink_data.xlsx
Nightingale lipid and metabolic data	This paper; GitHub	https://github.com/juliabrake/TANDEM/blob/main/TANDEM_Nightingale_data.xlsx

**Software and algorithms**		

R	The R Foundation for Statistical Computing	V.4.4.0

### Experimental model and study participant details

#### Study settings and participants

This study was performed as part of the TANDEM program.^48^ Study participants were recruited in Bandung City, West Java, Indonesia, from 2013 to 2017. Individuals with DM, TB or TB-DM were included as previously described.^49^^,^^50^ In short, people with previously diagnosed DM were recruited in the endocrine clinic at Hasan Sadikin Hospital and from 25 community health centers (CHCs). These individuals were screened for TB through a symptom assessment, which included questions about cough, fever, weight loss, dyspnea, chest discomfort, and sputum production. Additionally, a chest X-ray was conducted, and those with suggestive findings provided two sputum samples for acid-fast bacilli smear and *Mtb* culture. Individuals were classified as active TB-DM when TB symptoms, chest X-ray, *Mtb* culture, and smear result were positive.

Individuals with newly diagnosed pulmonary TB were recruited from two hospitals and 44 CHCs in Bandung. Individuals with known TB were screened for DM and classified as combined TB-DM when repeated glycated hemoglobin (HbA1c) was >6.5%. Exclusion criteria contained TB-treatment longer than 72 h, known HIV-positivity, corticosteroid use, hemoglobin <1 g/dL, emphysema, chronic bronchitis, asthma, pregnancy, other serious co-morbidity (such as cancer) or known alcohol abuse. Selection criteria for the study participants included the availability of plasma samples, and data on clinical characteristics, and treatment outcome. Additionally, efforts were made to ensure an even distribution of group sizes, sex, and age across the three phenotypes.

#### Ethics

The study received approval from the Observational Research Ethics Committee, London School of Hygiene and Tropical Medicine on December 18, 2013 (LSHTM ethics ref. 6449, LSHTM amendment no: A473) and the Health Research Ethics Committee, Faculty of Medicine, Universitas Padjadjaran, Indonesia (No. 05/UN6.C2.1.2/KEPK/PN/2014). All participants provided written informed consent.

### Method details

#### Clinical data and plasma collection

Clinical data for individuals with DM were collected, including the duration of the disease, medications, and complications. Anthropometric data (weight and height) were measured to calculate the body mass index (BMI). Blood samples were taken to measure HbA1c. For individuals with TB, additional clinical measurements were collected, including sputum culture results, chest X-ray scores, and hemoglobin levels. Xpert MTB/RIF test was performed for those suspected of having drug-resistant TB. Plasma samples were collected from random non-fasting blood samples according to standardized protocols provided by the consortium.

#### Inflammatory marker measurements

Circulating inflammatory markers were assessed using a commercially available multiplex proximity extension assay (Olink Proteomics, Uppsala, Sweden).^51^ In this assay, oligonucleotide-labeled antibodies bind to the protein, followed by linking of two antibodies when in close proximity, which is then detected and quantified via DNA polymerase extension and real-time PCR. To ensure accuracy, all samples were randomized across plates and plasma samples from the same participant at different time points were measured on the same plate to mitigate variations between plates. Normalization of detected proteins was performed based on inter-plate controls, and results were expressed as log2 transformed normalized protein expression (NPX) values. In total, 77 proteins were detectable in more than 75% of samples and thus included in the analysis. Measured samples were excluded when no protein was measurable (missing values) in all three phenotypes and both timepoints, hemolysate was >7.5 g/L, or when labeled with QC warning and simultaneous strong batch effect in the principal component analysis (PCA) analysis was seen.

#### Lipid and metabolite measurements

Plasma lipid measurement was performed using a high-throughput nuclear magnetic resonance spectroscopy platform (Nightingale Health, Helsinki, Finland), which gives absolute levels and compositions of lipids, lipoprotein subclasses, and additional metabolic markers. To focus on atherogenic lipids, 35 of the 225 measured markers were used for the main analysis. As for inflammatory markers, samples were randomized across plates. Measurements were excluded when the measured metabolite concentrations were under the lower limit of detection in more than 25% of the samples or when no metabolite was detectable (missing values) in all three phenotypes and both timepoints. Abbreviations and full names of all measured markers can be found in Table S1.

### Quantification and statistical analysis

Despite matching of the cohort groups, clinical characteristics were additionally tested for significant differences using a Wilcoxon rank-sum test (Mann-Whitney U test). Inflammatory protein concentrations were analyzed using the NPX values provided by Olink. Lipid measurements were analyzed using absolute concentrations provided by Nightingale. For all analyses, quality control was performed by excluding measurements with >25% missing values and by testing for the presence of batch effects using PCA. Measurements that passed quality control, were analyzed using PCA and clustering by z-scores of the median stratified by phenotype. Differences between groups were tested by using Wilcoxon rank-sum test (Mann-Whitney U test) (DM vs. TB, DM vs. TB-DM, TB vs. TB-DM) and illustrated in combined Volcano plots. Differences between timepoints were tested by a paired Wilcoxon signed-rank test (month 2 vs. baseline) for each group (TB, TB-DM). Log2 fold changes were used to visualize changes between timepoints. Associations with clinical characteristics were tested using linear regression and effect size calculation. Correlations between inflammatory markers and lipid measurements were tested using Spearman’s correlation. All results were assigned to be significant if the adjusted *p*-value was <0.05. All analyses were performed using R version 4.4.0. The entire data analysis workflow and code can be found in the Key resource table.

### Additional resources

This work is not part of or involving a clinical trial.
