# Supplementary material for: Inflammation and dyslipidaemia in combined diabetes and tuberculosis; a cohort study
Source: iScience. 2025 May 27;28(6):112760. doi: 10.1016/j.isci.2025.112760 (PMC12192345; doi:10.1016/j.isci.2025.112760)
Supplement: Document S1. Figures S1–S5 and Tables S1 and S2 [file mmc1.pdf]

iScience, Volume *28*

Supplemental information

Inflammation and dyslipidaemia

in combined diabetes and tuberculosis;

a cohort study

Julia Brake, Mandala Ajie, Nicholas A. Sumpter, Raspati C. Koesoemadinata, Nanny N.M. Soetedjo, Prayudi Santoso, Bachti Alisjahbana, Rovina Ruslami, Philip Hill, and Reinout van Crevel

S1 Table. Abbreviations of measured markers, Related to STAR methods.

Abbreviation	Full name
**4E-BP1**	Eukaryotic translation initiation factor 4E-binding protein 1
**ADA**	Adenosine deaminase
**ApoA1**	Apolipoprotein A1
**ApoB**	Apolipoprotein B
**AXIN1**	Axin-1
**CASP8**	Caspase-8
**CCL11**	Eotaxin
**CCL13**	C-C motif chemokine 13
**CCL19**	C-C motif chemokine 19
**CCL2**	C-C motif chemokine 2
**CCL20**	C-C motif chemokine 20
**CCL23**	C-C motif chemokine 23
**CCL25**	C-C motif chemokine 25
**CCL28**	C-C motif chemokine 28
**CCL3**	C-C motif chemokine 3
**CCL4**	C-C motif chemokine 4
**CCL7**	C-C motif chemokine 7
**CCL8**	C-C motif chemokine 8
**CD244**	Natural killer cell receptor 2B4
**CD274**	Programmed cell death 1 ligand 1
**CD40**	Tumor necrosis factor receptor superfamily member 5
**CD5**	T-cell surface glycoprotein CD5
**CD6**	T-cell differentiation antigen CD6
**CD8A**	T-cell surface glycoprotein CD8 alpha chain
**CDCP1**	CUB domain-containing protein 1
**Clinical LDL-C**	Clinical LDL cholesterol
**CSF-1**	Macrophage colony-stimulating factor 1
**CST5**	Cystatin-D
**CX3CL1**	Fractalkine
**CXCL1**	Growth-regulated alpha protein
**CXCL10**	C-X-C motif chemokine 10
**CXCL11**	C-X-C motif chemokine 11
**CXCL5**	C-X-C motif chemokine 5
**CXCL6**	C-X-C motif chemokine 6
**CXCL8**	Interleukin-8
**CXCL9**	C-X-C motif chemokine 9
**DNER**	Delta and Notch-like epidermal growth factor-related receptor
**EN-RAGE**	Extracellular newly identified receptor for advanced glycation end-products binding protein
**FGF19**	Fibroblast growth factor 19
**FGF-21**	Fibroblast growth factor 21
**FGF23**	Fibroblast growth factor 23
**FGF-5**	Fibroblast growth factor 5
**FLT3LG**	Fms-related tyrosine kinase 3 ligand
**GDNF**	Glial cell line-derived neurotrophic factor
**GlycA**	Glycoprotein acetyls
**HDL-C**	HDL cholesterol
**HDL-CE**	Cholesteryl esters in HDL
**HDL-FC**	Free cholesterol in HDL
**HDL-L**	Total lipids in HDL
**HDL-P**	Concentration of HDL particles
**HDL-PL**	Phospholipids in HDL
**HDL-TG**	Triglycerides in HDL
**HGF**	Hepatocyte growth factor
**IFNγ**	Interferon gamma
**IL-10**	Interleukin-10
**IL10-RA**	Interleukin-10 receptor subunit alpha
**IL10-RB**	Interleukin-10 receptor subunit beta
**IL-12B**	Interleukin-12 subunit beta
**IL15-RA**	Interleukin-15 receptor subunit alpha
**IL-17A**	Interleukin-17A
**IL-17C**	Interleukin-17C
**IL-18**	Interleukin-18
**IL18-R1**	Interleukin-18 receptor 1
**IL20-RA**	Interleukin-20 receptor subunit alpha
**IL-6**	Interleukin-6
**IL-7**	Interleukin-7
**KITLG**	Kit ligand
**LDL-C**	LDL cholesterol
**LDL-CE**	Cholesteryl esters in LDL
**LDL-FC**	Free cholesterol in LDL
**LDL-L**	Total lipids in LDL
**LDL-P**	Concentration of LDL particles
**LDL-PL**	Phospholipids in LDL
**LDL-TG**	Triglycerides in LDL
**LIFR**	Leukemia inhibitory factor receptor
**LTα**	Lymphotoxin-alpha
**MMP1**	Interstitial collagenase
**MMP-10**	Stromelysin-2
**non-HDL-C**	Total cholesterol minus HDL-C
**NRTN**	Neurturin
**NTF3**	Neurotrophin-3
**OSM**	Oncostatin-M
**PLAU**	Urokinase-type plasminogen activator
**Remnant-C**	Remnant cholesterol (non-HDL, non-LDL -cholesterol)
**S100A12**	Protein S100-A12
**SIRT2**	NAD-dependent protein deacetylase sirtuin-2
**SLAMF1**	Signaling lymphocytic activation molecule
**STAMBP**	STAM-binding protein
**SULT1A1**	Sulfotransferase 1A1
**TGFA**	Protransforming growth factor alpha
**TGF-β1**	Transforming growth factor beta-1 proprotein
**TNF**	Tumor necrosis factor
**TNFRSF11B**	Tumor necrosis factor receptor superfamily member 11B
**TNFRSF9**	Tumor necrosis factor receptor superfamily member 9
**TNFSF10**	Tumor necrosis factor ligand superfamily member 10
**TNFSF11**	Tumor necrosis factor ligand superfamily member 11
**TNFSF12**	Tumor necrosis factor ligand superfamily member 12
**TNFSF14**	Tumor necrosis factor ligand superfamily member 14
**TRAIL**	TNF-related apoptosis-inducing ligand
**TRANCE**	TNF-related activation-induced cytokine
**Total-C**	Total cholesterol
**Total-CE**	Total esterified cholesterol
**Total-FC**	Total free cholesterol
**Total-L**	Total lipids in lipoprotein particles
**Total-P**	Total concentration of lipoprotein particles
**Total-PL**	Total phospholipids in lipoprotein particles
**Total-TG**	Total triglycerides
**VEGFA**	Vascular endothelial growth factor A
**VLDL-C**	VLDL cholesterol
**VLDL-CE**	Cholesteryl esters in VLDL
**VLDL-FC**	Free cholesterol in VLDL
**VLDL-L**	Total lipids in VLDL
**VLDL-P**	Concentration of VLDL particles
**VLDL-PL**	Phospholipids in VLDL
**VLDL-TG**	Triglycerides in VLDL

S2 Table. Statistical comparison of patient characteristics, Related to Table 1. *

	DM vs. TB	DM vs. TB-DM	TB vs. TB-DM
**Age**	3.29e-09	0.25	2.12e-07
**Sex**	0.44	0.64	0.87
**BMI**	1.06e-23	6.32e-10	1.55e-05
**HbA1c**	3.76e-31	7.28e-06	1.88e-36
**Haemoglobin**	2.60e-10	7.35e-05	0.01
**Timika score**	NA	NA	0.15

* Values are presented as p-values. Statistical testing between two groups was done by either t-test for continuous variables or with Pearson's Chi-squared test with Yates' continuity correction for binary variables.

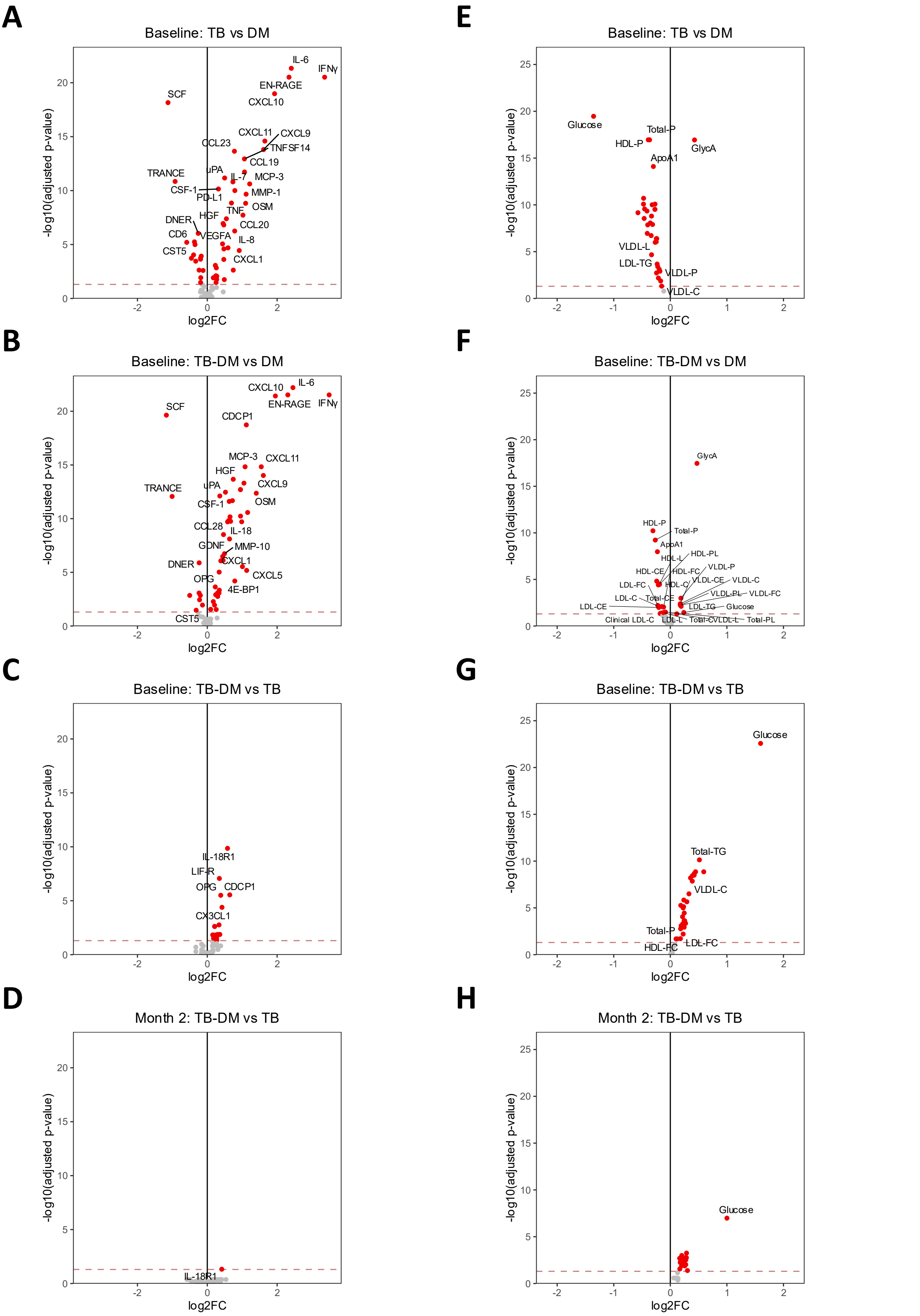

S1 Figure. Differences in inflammation and lipid markers between all groups and timepoints, Related to Figure 1-2 and 4-5. (A-D) Comparison of circulating inflammatory markers of individuals with DM (n = 96), TB (n = 93), and TB-DM (n = 91) at baseline. **(E-H)** Comparison of lipid intermediates and metabolic markers of individuals with DM (n = 93), TB (n = 91), and TB-DM (n = 83) at baseline. **(A-H)** Volcano plots showing significance ($-\log_{10}P$) versus magnitude of change (Log_2 fold change) of the differentially expressed markers. Significant differences between groups are depicted in red and nonsignificant differences are depicted in grey. Statistical testing was performed by Wilcoxon Rank Sum test (two-sided) between two groups.

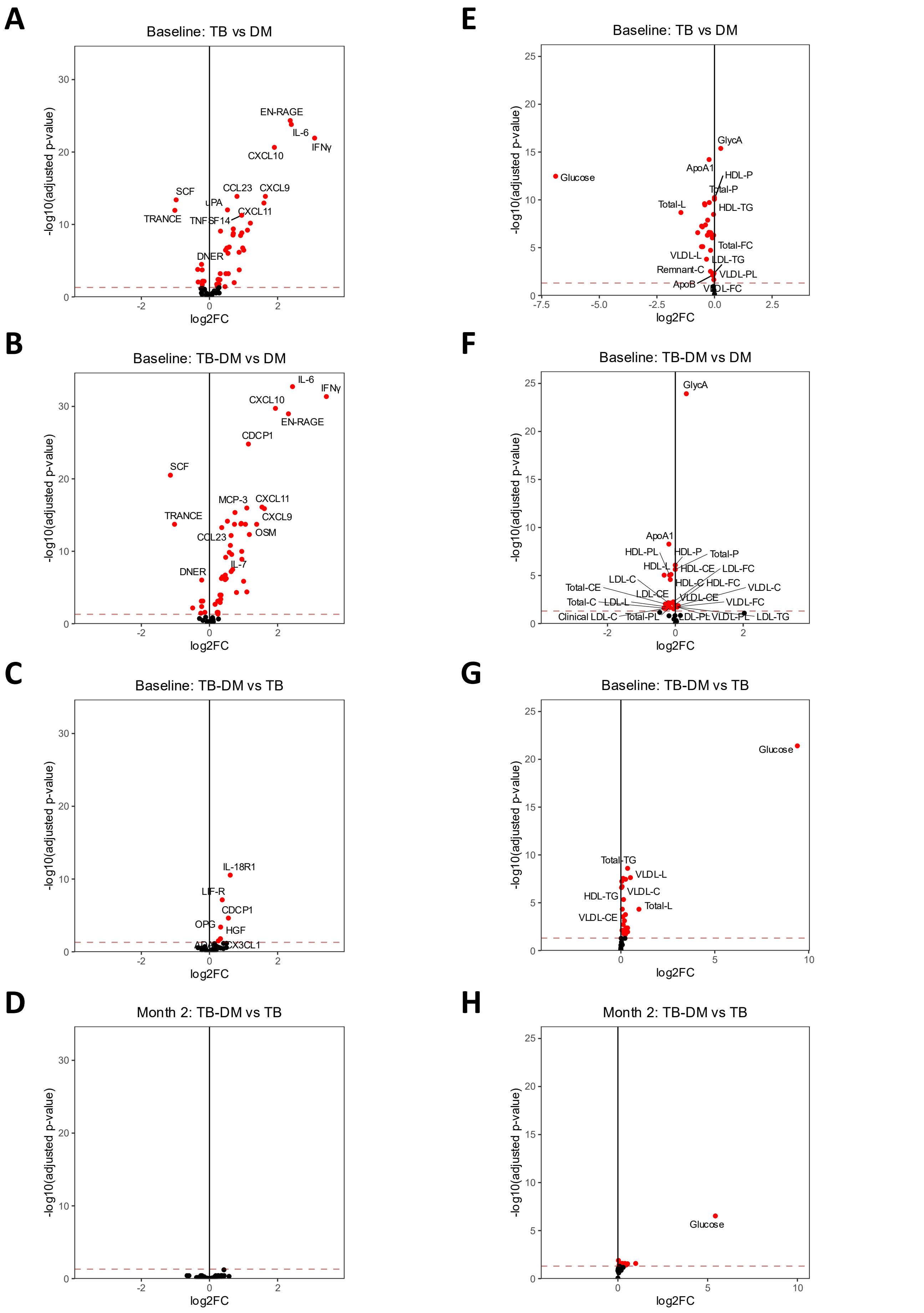

S2 Figure. Differences in inflammation and lipid markers between all groups and timepoints corrected for age and sex, Related to Figure 1-2 and 4-5. (A-D) Comparison of circulating inflammatory markers of individuals with DM (n = 96), TB (n = 93), and TB-DM (n = 91) at baseline. **(E-H)** Comparison of lipid intermediates and metabolic markers of individuals with DM (n = 93), TB (n = 91), and TB-DM (n = 83) at baseline. **(A-H)** Volcano plots showing significance (-$\log_{10}P$) versus magnitude of change (Log_2 fold change) of the differentially expressed markers. Significant differences between groups are depicted in red and nonsignificant differences are depicted in grey. Statistical testing was performed by linear regression between two groups corrected for age and sex.

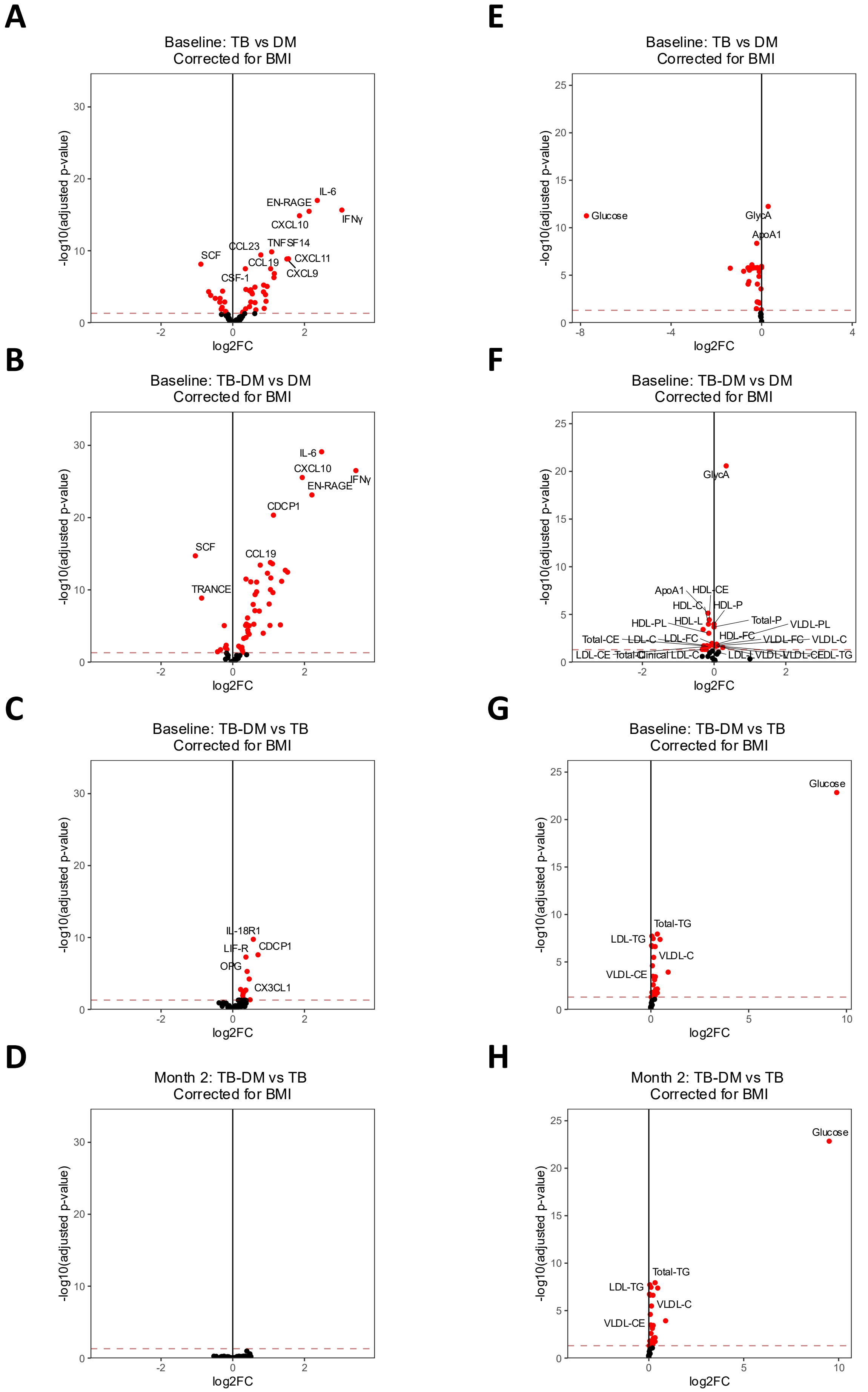

S3 Figure. Differences in inflammation and lipid markers between all groups and timepoints corrected for BMI, Related to Figure 1-2 and 4-5. (A-D) Comparison of circulating inflammatory markers of individuals with DM (n = 96), TB (n = 93), and TB-DM (n = 91) at baseline. **(E-H)** Comparison of lipid intermediates and metabolic markers of individuals with DM (n = 93), TB (n = 91), and TB-DM (n = 83) at baseline. **(A-H)** Volcano plots showing significance (-$\log_{10}P$) versus magnitude of change (Log_2 fold change) of the differentially expressed markers. Significant differences between groups are depicted in red and nonsignificant differences are depicted in grey. Statistical testing was performed by linear regression between two groups corrected for BMI.

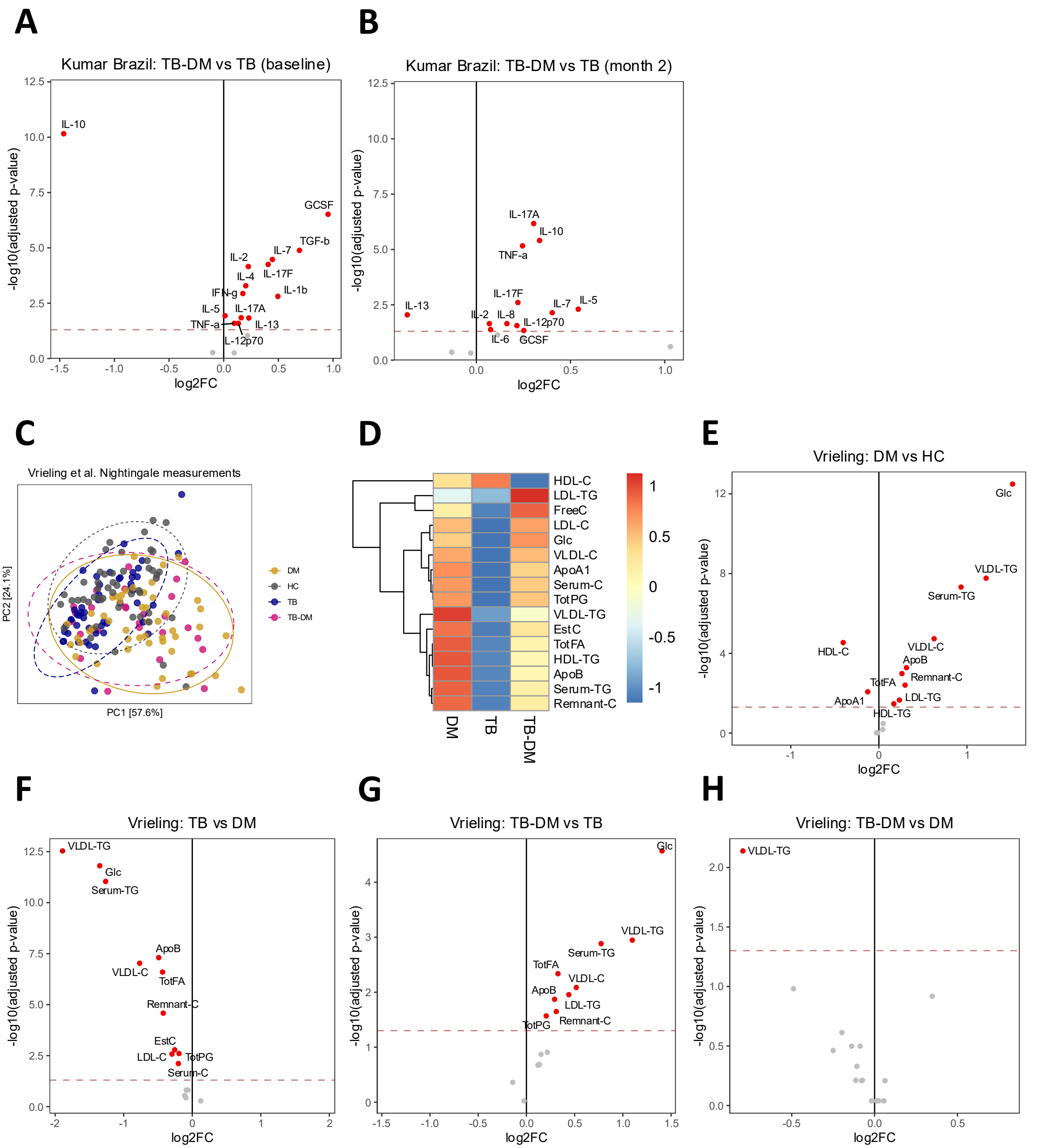

S4 Figure. Validation of inflammatory markers and lipid profiles in publicly available datasets, Related to Figure 1-2 and 4-5. (A-B) Comparison of cytokine levels in a Brazilian cohort from individuals with TB (n = 26) and TB-DM (n = 25) at **(A)** baseline and **(B)** after 2 months TB treatment. Volcano plots showing significance (-log10P) versus magnitude of change (Log2 fold change) of the differentially expressed markers. Significant differences between groups are depicted in red and nonsignificant differences are depicted in grey. Statistical testing was performed by Wilcoxon Rank Sum test (two-sided) between two groups. **(C-H) Comparison of** lipid levels at baseline in a South African cohort consisting of healthy individuals (HC = 50) or individuals with DM (n = 50), TB (n = 50), and TB-DM (n = 27). **(C)** Principal Component Analysis (PCA) plot shows the first two principal components derived from healthy (grey) or individuals with DM (yellow), TB (blue), and TB-DM (pink). **(D)** Heatmap representing the z-scores of the median of the groups for each measured marker. Higher and lower z-scores are depicted in red and blue, respectively. **(E-H)** Volcano plots showing significance (-log10P) versus magnitude of change (Log2 fold change) of the differentially expressed markers. Significant differences between groups are depicted in red and nonsignificant differences are depicted in grey. Statistical testing was performed by Wilcoxon Rank Sum test (two-sided) between two groups.

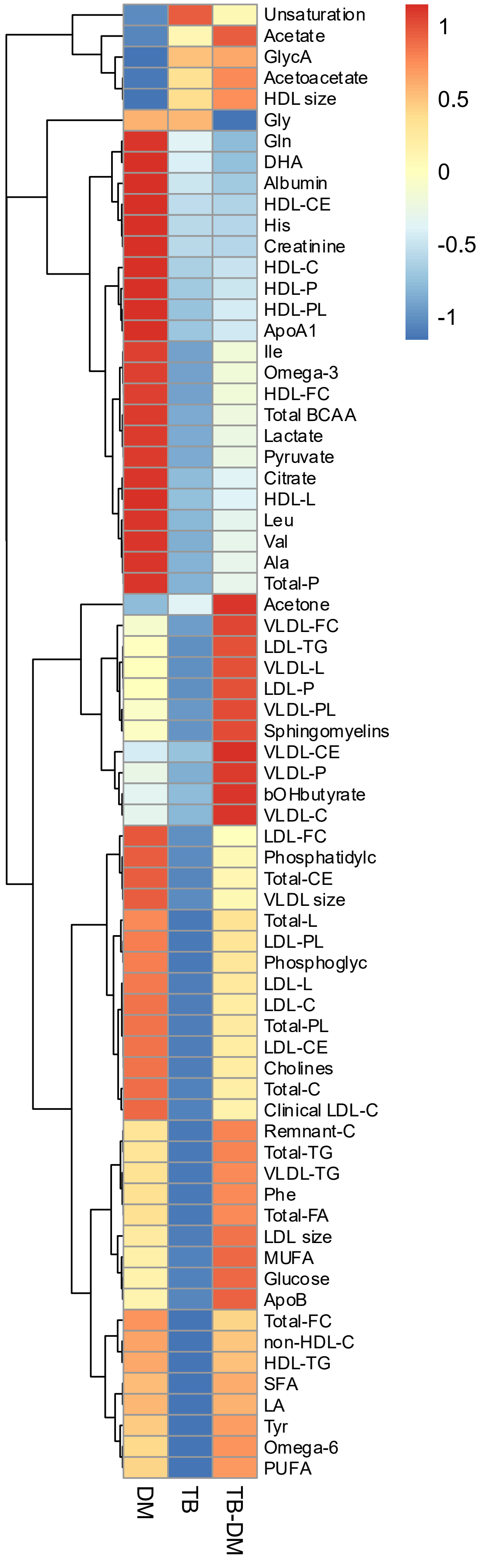

S5 Figure. Lipid intermediates and metabolic markers at baseline, Related to Figure 1-2 and 4-5. Comparison of all measured lipid intermediates and metabolic markers of individuals with DM (n = 93), TB (n = 91), and TB-DM (n = 83) at baseline. Heatmap representing the z-scores of the median of the three groups for each measured marker. Higher and lower z-scores are depicted in red and blue, respectively.
